# Catarrh of Pharynx

**Published:** 1890-08

**Authors:** 


CATARRH OF PHARYNX.—The following is claimed to be a useful gargle in catarrh of the pharynx:

| | |
|---|---|
| ℞—Sulph zinci | gr. xv. |
| Thymoli | gr. ⅓. |
| Alcoholis | āā f ʒ jss. |
| Glycerini puri | |
| Aq. Menth. pip. | f ℥ x. |
| M. | |

—*Medical and Surgical Reporter.*

